# The White Adrenal Line

**Published:** 1917-04

**Authors:** 


**The White Adrenal Line,** Emile Sergent, Paris, Endocrinology, Jan. 1917, marks some characteristic design on the abdomen, using the rounded end of a fountain pen or the finger tip, not the nail, and avoiding undue pressure or scratching. The test must not be made after the application of a poultice, compress, bandage, etc., and the clothing should have been removed at least 15 minutes before. The result should be viewed in a good light but shaded against glare. In well defined cases of adrenal insufficiency, a white line develops in about a minute and persists 1-3 minutes. The explanation is that in adrenal insufficiency there is general arterial hypotension, with peripheral vaso-dilatation and that light stimulation of the skin produces reflex vaso-constriction with blanching of the skin. The author warns against seeking absolute tests in general and one would imagine that this test would fail not only in cases of normal adrenal function lacking the initial superficial vaso-dilatation but in marked cases of failure of the adrenals in which even external stimulation would not produce a reflex constriction.

